# Assessment of renal impairment using estimated glomerular filtration rate among type 2 diabetes mellitus patients in North-East Ethiopia: a cross-sectional study

**DOI:** 10.1007/s40200-020-00680-4

**Published:** 2020-11-05

**Authors:** Mitku Mammo Taderegew

**Affiliations:** Department of Biomedical Sciences, School of Medicine, College of Medicine and Health Sciences, Wolkite University, P.O. Box 07, Wolkite, Ethiopia

**Keywords:** Renal impairment, Type 2 DM, Estimated glomerular filtration rate, Ethiopia

## Abstract

**Background:**

Chronic kidney disease (CKD) is the known cause of morbidity and mortality among diabetes mellitus (DM) patients. Targeted screening of renal impairment based on estimated glomerular filtration rate (eGFR) among DM patients has potential benefits in early identification and treatment of CKD. Hence, this study was aimed to estimate the magnitude of renal impairment using eGFR among type 2 DM patients.

**Methods:**

An institution-based cross-sectional study was conducted from February-1 to April 30/2020 among 422 type 2 DM patients in Northeast Ethiopia. Data were collected by the semi-structured questioner and serum creatinine measurement. The collected data were edited into Epi-data manager version 4.4.1.0, and the analysis was performed by SPSS-25. The Simplified Modification of Diet in Renal Disease (MDRD), Chronic Kidney Disease Epidemiology (CKD-EPI), and Cockcroft-Gault (C-G) equations were used to calculate eGFR.

**Results:**

Of all study participants, 82(19.4%), 92(21.8%), and 103(24.4%) had eGFR < 60 ml/min/1.73 m^2^, according to the MDRD, CKD-EPI, and C-G equations, respectively. Female sex, (MDRD:AOR = 4.44, 95%CI:1.97–9.97, CKD-EPI:AOR = 3.17, 95%CI:1.27–6.17, and C-G:AOR = 2.65, 95%CI:1.35–5.21), duration ≥ 10 years (MDRD:AOR = 3.38, 95%CI:1.45–7.92, CKD-EPI:AOR = 3.09, 95%CI:1.07–7.77, and C-G:AOR = 2.92, 95%CI:1.29–6.61), age ˃60 years (MDRD:AOR = 2.29, 95%CI:1.09–4.77, CKD-EPI:AOR = 4.12, 95%CI:1.68–6.78, and C-G: AOR = 3.42, 95%CI:1.77–6.60), hypertension (MDRD:AOR = 3.12, 95%CI:1.51–6.45, CKD-EPI: AOR = 4.21,95%CI:2.07–7.98, and C-G:AOR = 3.99, 95%CI:2.08–7.65), poor glycemic control (MDRD:AOR = 2.82, 95%CI:1.13–7.05, and C-G:AOR = 2.34, 95%CI:1.09–5.04), and body mass index (MDRD:AOR = 1.11, 95%CI:1.01–1.22, and CKD-EPI:AOR = 2.43, 95%CI:1.27–5.76) were significantly associated with renal impairment.

**Conclusion:**

Renal impairment was prevalent among type 2 DM patients. Older age, female sex, duration, hypertension, poor glycemic control, and BMI were significantly associated with renal impairment.

## Background

Chronic kidney disease (CKD) is a known public health problem worldwide with an estimated prevalence of 8–16% [1].

The prevalence of CKD is growing rapidly [1, 2] principally due to the growing prevalence of risk factors such as diabetes mellitus (DM) [3, 4]. DM, especially type 2 DM, is the foremost causes of CKD throughout the world [5–7]. Approximately 40–60% of individuals with type 2 DM had diabetic kidney disease [8, 9].

Due to the global epidemic of type 2 DM with disproportionate effects in the developing countries, the rate of diabetic kidney disease is growing rapidly in these countries [1, 2, 10]. Moreover, complications of DM are more common in developing countries because of shortage of screening and diagnostic resources [11–15]. This dual burden has led to a consequential increase in the number of type 2 DM patients with diabetic kidney disease without effective interventions [16, 17].

In Ethiopia, the prevalence of DM is growing alarmingly. In parallel, diabetes-related complications are rising. Additionally, in Ethiopia, renal complications of diabetes may go undetected due to limited diagnostic resources and late presentation of cases [18, 19].

Since diabetic kidney disease constitutes a major cause of chronic kidney disease, routine screening of renal impairment based on glomerular filtration rate among DM patients is therefore extremely important, as detecting chronic kidney disease during its initial stages provides the opportunity for early therapeutic interventions to slow its progression and to improve outcomes [20, 21].

Using serum creatinine alone is unreliable because its level is dependent on a number of factors that have nothing to do with a patient’s renal function. Instead an estimated glomerular filtration rate (eGFR) derived from prediction equations has been suggested as an important measure for assessing renal function [22, 23].

Increasing evidence suggests that reduced eGFR is a strong predictor of renal morbidity and mortality in DM patients [24, 25]. Furthermore, targeted screening of DM patients based on eGFR is cost-effective and has potential benefits in early identification and treatment of affected patients [24]. However, in clinical practice instead of using eGFR, direct measurement of serum creatinine level is used for assessing renal function test.

Thus, the finding of this study is helpful to establish the clinical relevance of providing eGFR for routine screening of renal impairment. The findings will also help the clinicians to appreciate the magnitude of renal impairment using eGFR among type 2 DM patients. It is also hoped that the study will provide an impetus for further research on renal complications in type 2 DM patients.

Hence, this study was designed to determine the magnitude of renal impairment among type 2 DM patients attending at public hospital in Northeast Ethiopia, based on eGFR derived from the Modification of Diet in Renal Disease (MDRD), Cockcroft-Gault (C-G), and Chronic Kidney Disease Epidemiology Collaboration (CKD-EPI) equations.

## Methods

### Study design, setting, and population

A Hospital-based cross-sectional study was conducted among type 2 DM patients from February-1 to April 30/2020. The study was undertaken among 422 type 2 DM patients who were registered at the follow-up clinic in Debre Berhan referral hospital, Northeast of Ethiopia.

All adults type 2 DM patients (≥ 18 years) who have a follow-up in the diabetic clinic in the study period were considered in the study, whereas patients diagnosed as having renal disease before a diagnosis of DM and diagnosis of a recent attack of acute kidney disease by medical experts, critically ill patients, dialysis patients, and under medications affecting kidney function were excluded from the study.

### Sample size determination

The required sample size of the study was estimated using a single population proportion formula, by considering: P = 50% (prevalence of expected renal impairment among type 2 DM patients), confidence interval (CI) of 95% (Z _a/2_= 1.96), and 5% margin of error. Then the minimum sample size obtained was 384. After adding a 10% non-response rate, a total of 422 type 2 DM patients were included in the study. A systematic random sampling technique based on participants’ order of flow (every third patient) was used for selecting the study participants. 

### Data collection procedures and laboratory measures

Socio-demographic, anthropometric, and clinical data were obtained with a questionnaire-based interview and from patient’s medical records during their medical examination. Their medical histories were interviewed and physical examinations include body weight, height, waist circumference (WC), and blood pressure were measured by nurses working in the clinic.

After calibrating the equipment’s and with patients being bare-footed and wearing light clothes, height and weight were measured using a stadiometer, and an electronic weight scale, respectively. Body mass index (BMI) was computed as weight (kg) divided by the square of the height (m^2^) as (kg/m^2^) and classified as: Normal weight (18–24.9 kg/m^2^), overweight (25–29.9 kg/m^2^), and obesity (≥ 30 kg/m^2^) [26]. WC was measured with the inelastic tape at midway between the lower margin of the last palpable rib and the top of the iliac crest. Central obesity was defined as a WC of ˃88 cm for females and ˃102 cm for males [﻿^27^, ﻿^28^]. All measurements were also taken three times and the mean values of three measurements were recorded for analysis.

Blood pressure was measured using an aneroid sphygmomanometer with an adult cuff size after 5 minutes of rest in sitting position in the right upper arm while placing the hand at the level of the heart. An average of three measurements with five minutes intervals between measurements was recorded. Participants were considered to have hypertension if systolic blood pressure (SBP) ≥ 130 mmHg and/or diastolic blood pressure (DBP) ≥ 80 or if they were taking any antihypertensive drugs [29].

Current smoker was defined as smoking one or more cigarettes per day in the last 3 months; former smoker: a subject who had stopped smoking before 3 months; nonsmoker: those had no habit of smoking [30].

Concerning glycemic control, four consecutive fasting blood sugar (FBS) measurements (at least 1-month interval between measurements) were taken from the patient’s medical records for calculating the mean FBS level. Then participants were classified as good glycemic control (average of four consecutive FBS were 80–130 mg/dl) and poor glycemic control (average of four consecutive FBS > 130 mg/dl mg/dl or < 80 mg/dl mg/dl ) [31].

Three milliliters of venous blood samples were collected with an aseptic venipuncture technique for serum creatinine analysis. After separating the serum, serum creatinine analysis was automated on ECHO XPC automatic chemistry analyzer (Edif instruments, Italy). All the participants were advised to have the other two serum creatinine checked-up within the next two months after the first check-up with keeping a 1-month interval with each check-up. Then three measurements with a 1-month interval were taken and the mean values of three measurements were recorded for analysis. Normal serum creatinine values were ≤ 1.5 mg/dl for males and ≤ 1.3 mg/dl for females [32].

### Measurement of kidney function

The estimated Glomerular Filtration Rate (eGFR) was estimated using the MDRD equation [33] as: eGFR = 186 × SCr (mg/dl)^−1.154^ × age(years)^−0.203^ × 0.742 (if female) × 1.210 (as our population are Africans), C-G equation as: eGFR=(140 - age) x body weight (kg) / 72 x SCr x 0.85 for female [34], and Chronic Kidney Disease Epidemiology Collaboration (CKD-EPI) equation as eGFR = 141 × min (Scr/κ, 1)^α^ x max (Scr/κ, 1)^−1.209^ x 0.993^age^ x 1.018 (if female) x 1.159 (as our participants black), where Scr is serum creatinine in mg/dL, κ is 0.7 for females and 0.9 for males, α is − 0.329 for females and − 0.411 for males, min indicates the minimum of Scr/κ or 1, and max indicates the maximum of Scr/κ or 1 [35]. Renal status was defined as normal or increased eGFR (if eGFR ≥ 90 ml/min/1.73 m^2^), mild, moderate, and severe renal impairment if eGFR was 60–89.9, 30–59.9, and, 15–29.9 ml/min/1.73 m^2^, respectively. For this study, renal impairment was considered as eGFR < 60 ml/min/1.73 m^2^.

### Statistical analysis

Before entry, data were checked, cleaned, coded, and sorted, for any lost values. Then data were edited into Epi-data manager version 4.4.1.0 and exported to statistical software for social sciences (SPSS) version 25 software. The data were also checked for its distribution and outliers before analysis. Then the data were processed by using descriptive analysis, including frequency tables, cross-tabulation, and summary measures. Categorical variables were stated as number (percentage) whereas the continuous data as means ± standard deviation (SD).

To determine the independently associated variables, associations were investigated using binary logistic regression analysis. All independent variables with p-value < 0.25 in the unadjusted model of logistic regression analysis were included in the multivariate model (backward stepwise) to control the effect of confounding variables and to identify independently associated factors with renal impairment in the final model. The degree of associations was expressed by using the odds ratio (ORs) with 95% CI. P-value with < 0.05 was considered statistically significant.

## Results

### Socio-demographic characteristics of the study participants

A total of 422 type 2 DM patients were invited and all of them were volunteered to participate in the study making the response rate of 100%. From the total of the study participants, 229 (54.3%) were female, and 260 (61.6%) were from urban areas. Their ages were ranged from 36 to 80 years old, with a mean (± SD) of 54.16 (± 10.61) years, with 302 (71.6%) of them aged below 60 years old. Nearly three fourth (73.7%) of the respondents were married, 206 (48.8%) of respondents attained high school and above, and 141 (33.4%) of respondents were government employees (Table 1).

**Table 1:** Socio-demographic characteristics of the study participants at public hospital in Northeast Ethiopia, 2020 (N = 422)

Variable	Category	Frequency (N = 422)	Percentage (%)
Sex	Male	193	45.7
	Female	229	54.3
Age (years)	< 60	302	71.6
	≥ 60	120	28.4
Marital Status	Single	45	10.6
	Married	311	73.7
	Divorced	29	6.9
	Widowed	37	8.8
Educational status	Illiterate	117	27.7
	Primary school (1–8)	99	23.5
	High school and above	206	48.8
Employment status	Farmer	96	22.7
	House wife	60	14.2
	Government Employee	141	33.4
	At private organization	107	25.4
	Others*	18	4.3
Residence	Urban	260	61.6
	Rural	162	38.4

Note: *Unemployed and students

### Clinical characteristics of the study participants

The mean (± SD) of DM duration was 6.2 (± 4.6) years with a minimum and maximum of 1 year to 24 years, respectively. Regarding anthropometric measurements, the mean (± SD) of BMI of the participants was 23.28 (± 3.64) Kg/m^2^ with 288 (68.2%) were classified as normal/underweight. About 91 (21.6%) of the study participants also had higher waist circumference for the gender-specific normal range.

The majority (82.9%) of the participants had no family history of kidney disease (FH-KD), 371 (87.9%) never smoked, and 396 (93.8%) had no history of cardiovascular disease. Current alcohol consumption habit was observed in 72 (17.1%) of participants. Regarding the treatment modality of the participants, nearly three fourth (73.9%) were taking oral hypoglycemic agent(s) only, while 110 (26.1%) were on a combination of insulin and oral hypoglycemic agents.

The mean systolic and diastolic blood pressures of participants were 133.69 (± 14.48) and 82.9 (± 11.10) mmHg, respectively. Nearly two fifths (39.1%) of participants were hypertensive with 140 (84.8%) of them were on antihypertensive treatments. More than three fifths (66.6%) of patients were presented with the level of poor glycemic control. Mean SCr (± SD) of the participants was 0.96 (± 0.32) mg/dl, and the majority of participants (88.9%) were with normal serum creatinine levels (Table 2).

**Table 2:** Clinical characteristics of study participants at public hospital in Northeast Ethiopia, 2020 (N = 422)

Characteristics	Categories	N (%)
Body mass index	Normal/underweight	288 (68.2)
	Overweight	78 (18.5)
	Obesity and above	56 (13.3)
Central obesity	Yes	91 (21.6)
	No	331 (78.4)
Duration of DM	< 10 Years	359 (85.1)
	≥ 10 Years	63 (14.9)
*SBP (mmHg)		133.69 ± 14.48
*DBP (mmHg)		82.90 ± 11.10
Hypertension	Yes	165 (39.1)
	No	257 (60.9)
Antihypertensive drug	None	282 (66.8)
	ACE-I or ARB	35 (8.3)
	ACE-I or ARB + Other	71 (16.8)
	Others	34 (8.1)
Serum creatinine (mg/dl)	Normal	375 (88.9)
	Higher	47 (11.1)
Glycemic control	Good	141 (33.4)
	Poor	281 (66.6)
Smoking status	Non-smoker	371 (87.9)
	Former smokers	32 (7.6)
	Current smoker	19 (4.5)

Note: ACE-I: Angiotensin-converting enzyme inhibitor; ARB: Angiotensin receptor blocker; DBP: Diastolic blood pressure; DM: Diabetes mellitus; SBP: Systolic blood pressure; *: data are expressed in Mean (± SD)

### Prevalence of renal impairment among the study participants

The mean eGFR (ml/min/1.73 m^2^) values of the participants estimated according to the MDRD, CKD-EPI, and C-G equations were 99.66 (± 34.24), 88.78 (± 22.32), and 84.87 (± 29.18) ml/min/1.73 m^2^, respectively.

In this study, the prevalence of renal impairment, defined as the presence of GFR < 60 ml/min/1.73 m^2^, were 19.4% (95%CI = 15.6% − 23.2%), 21.8% (95%CI = 17.9% − 25.7%), and 24.4% (95%CI = 20.3% − 28.5%), using the MDRD, CKD-EPI, and C-G equations, respectively. From those with renal impairment, 43.9%, 51.1%, and 57.3% of participants had normal serum creatinine level for MDRD, CKD-EPI, and C-G equation, respectively.

**Fig. 1:**
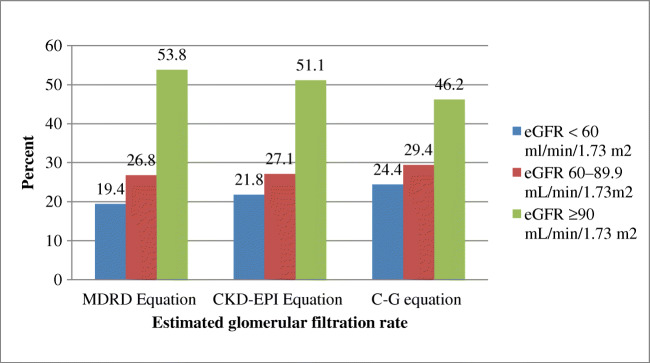
Prevalence of renal impairment among the study participants with MDRD, CKD-EPI, and C-G equation

Staging of renal impairments reveals that 227 (53.8%), 216 (51.2%, and 195 (46.2%) of the participants had eGFR of (≥ 90 mL/min/1.73 m^2^), while 113 (26.8%), 114 (27.0%), and 124 (29.4%) had eGFR of 60–89.9 mL/min/1.73 m^2^ in MDRD, CKD-EPI, and C-G equation, respectively. None of the participants had eGFR < 30 mL/min/1.73 m^2^ in all the three equations (Fig. 1).

### Factors associated with renal impairment among the study participants

In bivariable logistic regression analysis age ˃60 years, female sex, ≥ 10 years duration of DM, presence of hypertension, poor glycemic control, family history of kidney disease, increase in both systolic and diastolic blood pressures, and higher BMI were associated with the presence of renal impairments when renal function was assessed by using MDRD equation. Except for BMI, a similar pattern was found when the CKD-EPI and C-G equation was employed. Only independent variables with p-value < 0.25 in the unadjusted model of logistic regression analysis were included in the multivariate model.

In multivariable analysis, older age (AOR = 2.29, 95% CI: 1.09–4.77, P = 0.027), female sex (AOR = 4.44, 95% CI: 1.97–9.97; P < 0.001), long duration of DM (AOR = 3.38, 95% CI: 1.45–7.92, P = 0.005), poor glycemic control (AOR = 2.82, 95% CI: 1.13–7.05, P = 0.026), hypertension (AOR = 3.12, 95% CI: 1.51–6.45, P = 0.002) and increased BMI (AOR = 1.11, 95% CI: 1.01–1.22, P = 0.032) were significantly associated with the presence renal impairment (eGFR < 60 ml/min/1.73 m^2^) when the MDRD formula was used to assess renal function. When CKD-EPI equation was used age ˃60 years (AOR = 4.12, 95% CI: 1.68–6.78; P = 0.029), female sex (AOR = 3.17, 95% CI: 1.27–6.17; P = 0.001), ≥ 10 years duration of DM (AOR = 3.09, 95% CI: 1.07–7.77, P = 0.010), presence of hypertension (AOR = 4.21, 95% CI: 2.07–7.98, P < 0.001), and increased BMI (AOR = 2.43, 95% CI: 1.27–5.76, P = 0.018) were significantly associated with the presence of renal impairment.

According to C-G equation, age ˃60 years (AOR = 3.42, 95% CI: 1.77–6.60; P < 0.001), female sex (AOR = 2.65, 95% CI: 1.35–5.21; P = 0.005), ≥ 10 years duration of DM (AOR = 2.92, 95% CI: 1.29–6.61, P = 0.010), poor glycemic control (AOR = 2.34, 95% CI: 1.09–5.04, P = 0.029), and hypertension (AOR = 3.99, 95% CI: 2.08–7.65, P < 0.001) were significantly associated with the presence of renal impairment (Table 3).

**Table 3:** Multi-variable logistic regression of factors associated with renal impairment based on eGFR among type 2 DM patients attending at public hospital in Northeast Ethiopia, 2020 (N = 422)

Variables	MDRD equation		CKD-EPI equation		C-G equation	
	AOR (95%)	*P* value	AOR (95%)	*P* value	AOR (95%)	*P*-value
Sex						
Male	1.00		1.00		1.00	
Female	4.44 (1.97–9.97)	0.001*	3.17 (1.27–6.17)	0.001*	2.65 (1.35–5.21)	0.005*
Age						
≤60	1.00		1.00		1.00	
>60	2.29 (1.09–4.77)	0.027*	4.12 (1.68–6.78)	0.029*	3.42 (1.77–6.60)	0.001*
Duration of DM						
< 10 Years	1.00		1.00		1.00	
≥ 10 Years	3.38 (1.45–7.92)	0.005*	3.09 (1.07–7.77)	0.010*	2.92 (1.29–6.61)	0.010*
Hypertension						
Yes	3.12 (1.51–6.45)	0.002*	4.21 (2.07–7.98)	0.001*	3.99 (2.08–7.65)	0.001*
No	1.00		1.00		1.00	
Family history of kidney disease						
Yes	1.06 (0.98–1.14	0.095	2.01 (0.27–5.37)	0.118	2.13 (0.37–4.27)	0.127
No	1.00		1.00		1.00	
Glycemic control						
Good	1.00		1.00		1.00	
Poor	2.82 (1.13–7.05)	0.026*	2.57 (0.61–8.92)	0.172	2.34 (1.09–5.04)	0.029*
BMI**	1.11 (1.01–1.22)	0.032*	2.43 (1.27–5.76)	0.018*	0.95 (0.86–1.04)	0.270
SBP**	0.98 (0.64–1.02)	0.236	0.78 (0.49–1.07)	0.317	0.97 (0.71–1.01)	0.056
DBP**	1.01 (0.95–1.06)	0.980	1.02 (0.97–1.08)	0.099	1.05 (0.99–1.12)	0.064

Note: AOR: Adjusted odds ratio; BMI: Body mass index; C-G: Cockcroft–Gault; CKD-EPI: Chronic Kidney Disease; N: Number; MDRD: Modification of Diet in Renal Risease; 1.00: Reference group; * indicates the level of significance (*p* < 0.05). **: data are expressed in Mean (± SD)

## Discussion

In this hospital-based cross-sectional study, the eGFR among type 2 DM patients at public hospital in Northeast Ethiopia has been assessed, using MDRD, CKD-EPI, and C-G equation. It was found that the prevalence of renal impairment was 19.4%, 21.8%, and 24.3% in MDRD, CKD-EPI, and C-G equation, respectively.

The prevalence of renal impairment using the MDRD equations (19.4%) was comparable with the study conducted in Australia (23.1%) [36], and United Arab Emirate (22.3%) [37] but lower than studies from Singapore [38], Netherland [39], and Brazil [20] where the prevalence of renal impairment has ranged from 25–53%. The magnitude of renal impairment was higher than the findings in Gondar (14.3%) [40] and Nigeria (11.4%) [41] but relatively lower than the findings in Kenya (38.6%) [42] and Bangladesh (54.5%) [43] when eGFR was assessed with CKD-EPI equation. These variations in the prevalence of renal impairment between this study and other studies might be expressed by the differences in study designs, socio-demographic and clinical characteristics of the study participants and differences in ethnic background of the study participants.

When the renal function was assessed using the C-G equation, the magnitude of renal impairment (24.3%) was in line with the study in Tanzania (24.7%) [44], Thailand (27.09%)[9] and Brazil (25.3%) [20]. However, this finding was relatively higher than the study in Sweden (7%) [30]. These variations might also be due to differences in ethnicity, healthcare setting, socio-demographic characteristics, and methodology applied [45].

Indeed, the SCr measurement is the most common method for assessing kidney function, use of creatinine alone can show the function of the kidney in many diabetic patients falsely normal [20, 46]. In DM patients, the eGFR usually will be less than half of normal before the serum creatinine increase above the laboratory normal range [47]. Considering the percentage of renal impaired patients with normal serum creatinine levels in this study, the above description seems acceptable. If the decreased kidney function was assessed based on elevated serum creatinine, renal impairment was found only in 11.1% of these type 2 DM patients. As the result showed screening techniques based upon abnormal serum creatinine would fail to detect a significant number of subjects with an eGFR < 60 ml/min/1.73 m^2^. Therefore, without measuring eGFR the clinician may not be informed of the existence of renal impairment and be wrongly reassured that renal function is well.

In this study, we found that using the C-G equation estimate a higher magnitude of renal impairment (24.3%) than the MDRD and CKD-EPI equations, which estimate19.4% and 21.8%, respectively. C-G equation does not incorporate race, which may influence creatinine excretion [48] might be the possible reason for the high prevalence of renal impairment in the C-G equation. Whatever the reason, C-G may help in early identification and proper categorization of renal impairment, which may allow for timely and specific interventional therapy to prevent further progression of kidney disease and its related complications.

In this study, gender difference has a significant association with renal impairments when renal function was assessed by the three equations in which the female gender was reported to be a non-avoidable risk factor for renal impairment. This is in agreement with the findings in Sweden (eGFR; MDRD and C-G) [30] and UK (eGFR; MDRD) [49] but unlike with the study conducted in Belgium (eGFR; only in MDRD) [50]. This difference may be attributed to a greater number of younger obese men in these study participants.

In this study, age was significantly associated with the presence of renal impairments, in which participants with > 60 years were more likely to have renal impairment based on MDRD, CKD-EPI, and C-G equations. This is in line with previous reports of the increasing prevalence of kidney disease with an increase in age in the Netherland [39], Sweden [30], and Singapore [38].

As age increases, there is gradual damage of nephrons mainly due to comorbidities that are common at the elderly, such as, hypertension, obesity, and decreased renal blood flow, which leads to chronic kidney disease [51, 52]. Type 2 DM patients with old age may also have vascular and tubulointerstitial changes due to the existence of potential senescence of glomeruli due to aging itself [2].

According to our study, the presence of hypertension among type 2 DM patients was associated with the development of renal impairment in MDRD, CKD-EPI, and C-G equation. This was in agreement with other related studies, which showed a significant association between being hypertension and renal impairments [9, 10, 30, 38].

In hypertensive patients, big arteries lose their compliance and increase its stiffness, which resulted in less safeguarding of blood pressure variation and wider blood pressure fluctuations for any given change in the stroke volume. The oscillatory shear stress resulted from this wider blood pressure fluctuations causes the development and progression of atherosclerosis of the renal vasculature which could contribute to the impairment of renal function [10, 53].

Based on MDRD, and C-G equation, in line with other study findings [20, 38, 50] our results showed a significant association between glycemic control and the presence of renal impairments in which patients with poor glycemic control were more likely to have renal impairments. However, in contrast with our findings, the study in the United Kingdom [49] reported a negative association between glycemic control and renal impairment.

In poor glycemic control, chronic hyperglycemia has been found to play a key role in decreasing eGFR and accelerating the annual eGFR decline. The cellular mechanisms responsible for hyperglycemia-induced renal damage include the formation of advanced glycation end products (AGEs) and activation of their receptor, thereby generation of reactive oxygen species (ROS) and a chronic subacute inflammatory process which play a pivotal role in the development of chronic kidney disease. Also, activation of AGEs receptors’ induces glomerular matrix production and increases oxidative stress, which promotes epithelial-mesenchymal transdifferentiation of renal tubular cells, thus contributing to interstitial fibrosis and renal histological change [54].

It was also likely that the difference in the prevalence of renal impairments might result from differences in the duration of DM using the MDRD, CKD-EPI, or C-G equation. Compared to respondents with a short duration of DM, respondents with a long duration had greater odds for the occurrence of renal impairments. This corresponds with the findings of several studies that reported that the possibility of developing renal impairment was greater among patients with longer duration of DM [9, 30].

In this study, BMI has a significant association with renal impairments when renal function was assessed by both MDRD and CKD-EPI equation. The results of this study were in agreement with various studies [30, 38] which showed that high BMI was associated with renal impairment. Suggested pathways of obesity include insulin resistance, low-grade inflammation, and endothelial dysfunction, which have been suggested as potential mechanisms for the development of renal impairment [55, 56].

Anatomical and physiological changes related to obesity including an increase in glomerular size, increased mesangial matrix and mesangial cell proliferation, hypertrophy and effacement of podocytes, and increase growth factors and adipokine alterations which may lead to fibrosis were also another possible mechanism for renal impairments in obese individuals [57].

Unlike a study conducted in Sweden [30] in which low BMI is associated with the development of renal impairment, in this study there was no statistically significant association when renal function was estimated using the C–G equation. This variation may be due to the difference in the distribution of obesity among the study participants. In obese subjects, creatinine production rate related to the body weight is less than normal-weight subjects. Because of the presence of weight in the C-G formula, using serum creatinine to calculate GFR by C-G formula may be higher than normal [9].

The strength of this study is that it is one of the few studies with including the three equations for estimating GFR from serum creatinine level.

The study has its limitations that need to be considered. The uses of eGFR rather than direct measured GFR for assessing renal impairment, use of FBS instead of HbA1c for assessing the level of glycemic status, and due to selection bias during selecting participants there may be reduction in the external validity. Due to limited resources, the non-diabetic renal diseases of course may also coexist the study participants were not excluded and the equation used for estimating GFR was not validated among adult populations of Ethiopian origin were also the limitation of the study.

## Conclusions

The prevalence of renal impairment was 19.4%, 21.8%, and 24.3% when using MDRD, CKD-EPI, and C-G equations, respectively but only 11.1% would have been classified as having renal impairment if SCr used alone for assessing kidney status. Older age, female sex, longer duration of DM, hypertensive, increased BMI, and poor glycemic control were significantly associated with renal impairment. Physicians should not depend on serum creatinine only as a measure of kidney function instead use the serum creatinine to estimate eGFR and assessing of renal impairment. Thus, early screening based on eGFR would allow more aggressive measures to be taken to prevent further progression and adverse outcomes.

## References

[1] Mills KT, Xu Y, Zhang W, Bundy JD, Chen CS, Kelly TN (2015). A systematic analysis of worldwide population-based data on the global burden of chronic kidney disease in 2010. Kidney Int.

[2] Kramer H, Molitch ME (2005). Screening for kidney disease in adults with diabetes. Diabetes Care.

[3] Wachukwu CM, Emem-Chioma PC, Wokoma FS, Oko-Jaja RI (2015). Prevalence of risk factors for chronic kidney disease among adults in a university community in southern Nigeria. Pan Afr Med J..

[4] Barsoum RS (2006). Chronic kidney disease in the developing world. New Engl J Med.

[5] Kovesdy CP, Sharma K, Kalantar-Zadeh K (2008). Glycemic control in diabetic CKD patients: where do we stand?. American journal of kidney diseases.

[6] Gaballa MR, Farag YM. Predictors of diabetic nephropathy. Central European Journal of Medicine. 2013;8(3):287–96.

[7] Park J, Lertdumrongluk P, Molnar MZ, Kovesdy CP, Kalantar-Zadeh K (2012). Glycemic control in diabetic dialysis patients and the burnt-out diabetes phenomenon. Curr Diabetes Rep.

[8] American Diabetes Association. Standards of Medical Care in diabetes. J Clin Appl Res Educ. 2015;38, supple (October 2012):s1–94. Available from http://www.bvs.hn/Honduras/UICFCM/Diabetes/Diabetes.Care-1.pdf. Accessed 21 Jan 2020.

[9] Narenpitak S, Narenpitak A (2008). Prevalence of chronic kidney disease in type 2 diabetes in primary health care unit of Udon Thani province, Thailand. J Med Assoc Thail.

[10] Yeh CH, Yu HC, Huang TY, Huang PF, Wang YC, Chen TP (2017). The risk of diabetic renal function impairment in the first decade after diagnosis of diabetes mellitus is correlated with high variability of visit-to-visit systolic and diastolic blood pressure: a case-control study. BMC Nephrol.

[11] Thomas MC, Cooper ME, Zimmet P (2016). Changing epidemiology of type 2 diabetes mellitus and associated chronic kidney disease. Nat Rev Nephrol..

[12] Daar AS, Singer PA, Persad L, Pramming SK, Matthews R, Beaglehole R (2007). Grand challenges in chronic non-communicable diseases. Nature.

[13] Choukem SP, Fabreguettes C, Akwo E, Porcher R, Nguewa JL, Bouche C (2014). Influence of migration on characteristics of type 2 diabetes in sub-Saharan Africans. Diabetes Metab.

[14] Gill GV, Mbanya JC, Ramaiya KL, Tesfaye S (2009). A sub-Saharan African perspective of diabetes. Diabetologia.

[15] Gill G, Gebrekidan A, English P, Wile D, Tesfaye S (2008). Diabetic complications and glycaemic control in remote North Africa. QJM..

[16] Wang V, Vilme H, Maciejewski ML, Boulware LE. The economic burden of chronic kidney disease and end-stage renal disease. Semin Nephrol. 2016;36(4):319–30. 10.1016/j.semnephrol.2016.05.008. 10.1016/j.semnephrol.2016.05.008 27475662

[17] Naicker S. Burden of end-stage renal disease in sub-Saharan Africa. Clin Nephrol. 2010;74:13–6. 10.5414/cnp74s013. 10.5414/cnp74s013 20979956

[18] Guariguata L, Whiting DR, Hambleton I, Beagley J, Linnenkamp U, Shaw JE (2014). Global estimates of diabetes prevalence for 2013 and projections for 2035. Diabetes Res Clin Pract.

[19] Abebe N, Kebede T, Addise D (2017). Diabetes in Ethiopia 2000–2016–prevalence and related acute and chronic complications; a systematic review. Afr J Diabetes Med.

[20] Fontela PC, Winkelmann ER, Ott JN, Uggeri DP (2014). Estimated glomerular filtration rate in patients with type 2 diabetes mellitus. Rev Assoc Med Bras (1992).

[21] Fiseha T, Kassim M, Yemane T. Chronic kidney disease and underdiagnosis of renal insufficiency among diabetic patients attending a hospital in Southern Ethiopia. *B*MC Nephrol. 2014;15:198. Published 2014 Dec 15. 10.1186/1471-2369-15-198. 10.1186/1471-2369-15-198 PMC4277829 25511372

[22] Kalyesubula R, Fabian J, Nakanga W, Newton R, Ssebunnya B, Prynn J (2020). How to estimate glomerular filtration rate in sub-Saharan Africa: design and methods of the African Research into Kidney Diseases (ARK) study. BMC Nephrol.

[23] Boettcher C, Utsch B, Galler A, Grasemann C, Borkenstein M, Denzer C (2020). Estimated glomerular filtration rates calculated by new and old equations in children and adolescents with type 1 diabetes—What to do with the results?. Front Endocrinol.

[24] Manns B, Hemmelgarn B, Tonelli M, Au F, Chiasson TC, Dong J (2010). Population-based screening for chronic kidney disease: a cost-effectiveness study. BMJ.

[25] McGovern AP, Rusholme B, Jones S, van Vlymen JN, Liyanage H, Gallagher H (2013). Association of chronic kidney disease (CKD) and failure to monitor renal function with adverse outcomes in people with diabetes: a primary care cohort study. BMC Nephrol.

[26] Seidell JC, Flegal KM (1997). Assessing obesity: classification and epidemiology. Br Med Bull.

[27] Guerra RS, Amaral TF, Marques EA, Mota J, Restivo MT. Anatomical location for waist circumference measurement in older adults: a preliminary study. Nutr Hosp. 2012;27(5):1554–61. 10.3305/nh.2012.27.5.5922 23478705

[28] Consultation WHO. Waist circumference and waist-hip ratio. Report of a WHO Expert Consultation. Geneva: World Health Organization; 2008. pp. 8–11.

[29] Whelton PK, Carey RM, Aronow WS, Casey DE, Collins KJ, Himmelfarb CD (2018). 2017 ACC/AHA/AAPA/ABC/ACPM/AGS/APhA/ASH/ASPC/NMA/PCNA guideline for the prevention, detection, evaluation, and management of high blood pressure in adults: a report of the American College of Cardiology/American Heart Association Task Force on Clinical Practice Guidelines. J Am Coll Cardiol.

[30] Afghahi H, Cederholm J, Eliasson B, Zethelius B, Gudbjrnsdottir S, Hadimeri H (2011). Risk factors for the development of albuminuria and renal impairment in type 2 diabetes—the Swedish National Diabetes Register (NDR). Nephrol Dial Transplant.

[31] Kassahun T, Eshetie T, Gesesew H (2016). Factors associated with glycemic control among adult patients with type 2 diabetes mellitus: A cross-sectional survey in Ethiopia. BMC Res Notes.

[32] Fiseha T, Mengesha T, Girma R, Kebede E, Gebreweld A (2019). Estimation of renal function in adult outpatients with normal serum creatinine. BMC Res Notes.

[33] Levey AS, Bosch JP, Lewis JB, Greene T, Rogers N, Roth D (1999). A more accurate method to estimate glomerular filtration rate from serum creatinine: a new prediction equation. Ann Intern Med.

[34] Cockcroft DW, Gault MH (1976). Prediction of creatinine clearance from serum creatinine. Nephron.

[35] Levey AS, Stevens LA, Frcp C, Schmid CH, Zhang YL, Iii AFC (2009). A new equation to estimate glomerular filtration rate. Ann Intern Med.

[36] Thomas MC, Weekes AJ, Broadley OJ, Cooper ME, Mathew TH (2006). The burden of chronic kidney disease in Australian patients with type 2 diabetes (the NEFRON study). Med J Aust.

[37] Jamal Shahwan M, Hassan NAG, Shaheen RA (2019). Assessment of kidney function and associated risk factors among type 2 diabetic patients. Diabetes Metab Syndr.

[38] Low SK, Sum CF, Yeoh LY, Tavintharan S, Ng XW, Lee SB (2015). Prevalence of chronic kidney disease in adults with type 2 diabetes mellitus. Ann Acad Med Singap.

[39] Van der Meer V, Wielders HP, Grootendorst DC, de Kanter JS, Sijpkens YW, Assendelft WJ (2010). Chronic kidney disease in patients with diabetes mellitus type 2 or hypertension in general practice. Br J Gen Pract..

[40] Alemu H, Hailu W, Adane A. Prevalence of chronic kidney disease and associated factors among patients with diabetes in Northwest Ethiopia: A hospital-based cross-sectional study. Curr Ther Res Clin Exp. 2020;92:100578. Published 2020 Feb 26. 10.1016/j.curtheres.2020.100578. 10.1016/j.curtheres.2020.100578 PMC7068620 32190131

[41] Ulasi II, Ijoma CK, Onodugo OD, Arodiwe EB, Ifebunandu NA, Okoye JU (2013). Towards prevention of chronic kidney disease in Nigeria: a community-based study in Southeast Nigeria. Int Soc Nephrol.

[42] Mwenda V, Githuku J, Gathecha G, Wambugu BM, Roka ZG, Ong’or WO. Prevalence and factors associated with chronic kidney disease among medical inpatients at the Kenyatta National Hospital, Kenya, (2018). A cross-sectional study. Pan Afr Med J.

[43] Rahim MA, Mitra P, Haque HF, Samdani TS, Zaman S, Uddin KN (2017). Prevalence of chronic kidney disease stages 3–5 among patients with type 2 diabetes mellitus in Bangladesh. IMC J Med Sci.

[44] Janmohamed MN, Kalluvya SE, Mueller A, Kabangila R, Smart LR, Downs JA (2013). Prevalence of chronic kidney disease in diabetic adult out-patients in Tanzania. BMC Nephrol.

[45] Nair S, Mishra V, Hayden K, Lisboa PJ, Pandya B, Vinjamuri S (2011). The four-variable modification of diet in renal disease formula underestimates glomerular filtration rate in obese type 2 diabetic individuals with chronic kidney disease. Diabetologia.

[46] McFarlane P, Cherney D, Gilbert RE, Senior P, Diabetes Canada Clinical Practice Guidelines Expert Committee (2018). Chronic kidney disease in diabetes. Can J Diabetes.

[47] Thomas D, Zachariah S, Elamin AE, Hashim AL (2017). Limitations of serum creatinine as a marker of renal function. Sch Acad J Pharm.

[48] Launay-Vacher V, Chatelut E, Lichtman SM, Wildiers H, Steer C, Aapro M (2007). Renal insufficiency in elderly cancer patients: International Society of Geriatric Oncology clinical practice recommendations. Ann Oncol.

[49] Middleton RJ, Foley RN, Hegarty J, Cheung CM, Mcelduff P, Gibson JM (2006). The unrecognized prevalence of chronic kidney disease in diabetes. Nephrol Dial Transplant.

[50] Goderis G, Van Pottelbergh G, Truyers C, Van Casteren V, De Clercq E, Van Den Broeke C (2013). Long-term evolution of renal function in patients with type 2 diabetes mellitus: a registry-based retrospective cohort study. BMJ Open.

[51] Glassock RJ, Winearls C (2009). Ageing and the glomerular filtration rate: truths and consequences. Trans Am Clin Climatol Assoc.

[52] Eriksen BO, Tomtum J, Ingebretsen OC (2010). Predictors of declining glomerular filtration rate in a population-based chronic kidney disease cohort. Nephron Clin Pract.

[53] Chappell DC, Varner SE, Nerem RM, Medford RM, Alexander RW (1998). Oscillatory shear stress stimulates adhesion molecule expression in cultured human endothelium. Circ Res.

[54] MacIsaac RJ, Jerums G, Ekinci EI (2017). Effects of glycemic management on diabetic kidney disease. World J Diabetes.

[55] Kobayashi H, Tokudome G, Hara Y, Sugano N, Endo S, Suetsugu Y, Kuriyama S, Hosoya T (2009). Insulin resistance is a risk factor for the progression of chronic kidney disease. Clin Nephrol.

[56] Persson F, Rossing P, Hovind P, Stehouwer CDA, Schalkwijk CG, Tarnow L (2008). Endothelial dysfunction and inflammation predict the development of diabetic nephropathy in the Irbesartan in patients with type 2 diabetes and microalbuminuria (IRMA 2) study. Scand J Clin Lab Investig.

[57] Kopple JD, Feroze U. The effect of obesity on chronic kidney disease. J Ren Nutr. 2011;21(1):66–71. 10.1053/j.jrn.2010.10.009. 10.1053/j.jrn.2010.10.009 21195923

